# Supplementary material for: Polyglucose nanoparticles with renal elimination and macrophage avidity facilitate PET imaging in ischaemic heart disease
Source: Nat Commun. 2017 Jan 16;8:14064. doi: 10.1038/ncomms14064 (PMC5241815; doi:10.1038/ncomms14064)
Supplement: Supplementary Information — Supplementary Figures [file ncomms14064-s1.pdf]

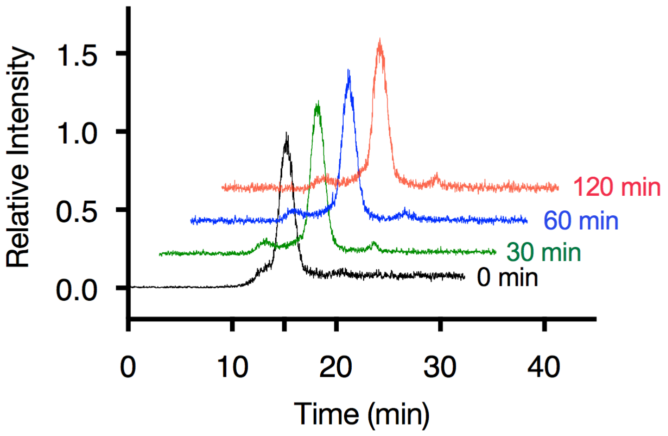

Supplementary Figure 1. Stability of Macroflor in mouse serum. Macroflor was incubated in mouse serum at 37 ºC for 120 min, and samples at each time point were injected to radio-size exclusion chromatography. Data are normalized to relative intensity.

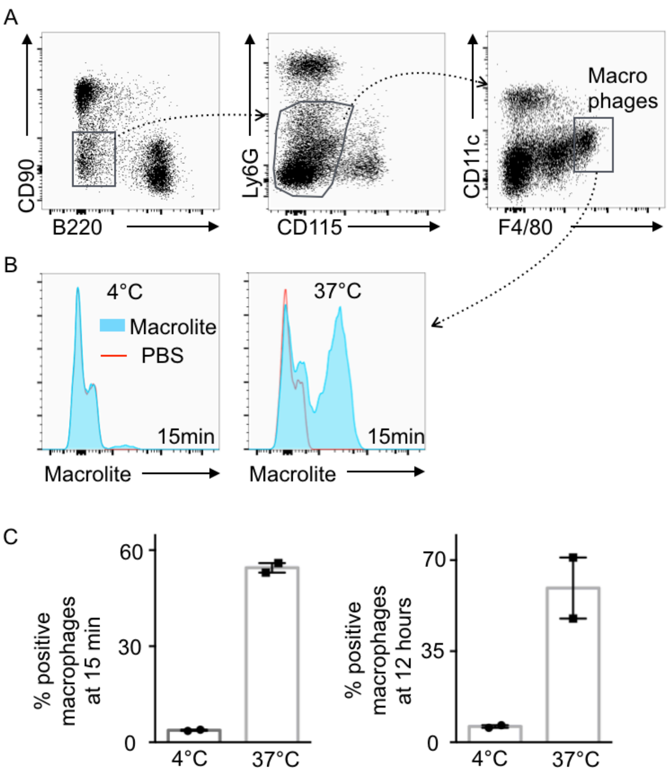

Supplementary Figure 2. Flow cytometry of splenocytes incubated in vitro with Macrolite at 4°C and 37°C. (A) Flow cytometric gating on splenocytes. (B) Histograms of Macrolite fluorescent intensity in macrophages after incubation at 4°C and 37°C. (C) Bar graphs show percentages of Macrolite positive macrophages incubated for 15 minutes and 12 hours at the two temperatures. Experiment was done in duplicate cell populations.

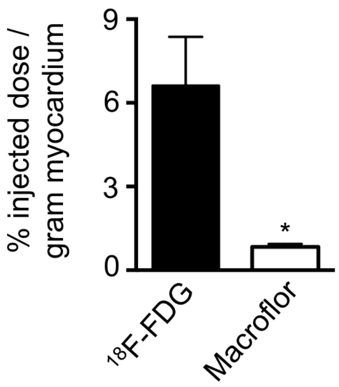

Supplementary Figure 3. Scintillation counting of healthy myocardium in wild type mice 3 hours after injection of ^{18}F-FDG versus Macroflor (Mean± SEM, n=7-8, *p<0.01, two-tailed t test).
